# Perspectives From Systems Biology to Improve Knowledge of *Leishmania* Drug Resistance

**DOI:** 10.3389/fcimb.2021.653670

**Published:** 2021-04-30

**Authors:** Elvira Cynthia Alves Horácio, Jéssica Hickson, Silvane Maria Fonseca Murta, Jeronimo Conceição Ruiz, Laila Alves Nahum

**Affiliations:** ^1^ René Rachou Institute, Oswaldo Cruz Foundation, Belo Horizonte, Brazil; ^2^ Department of Genetics, Ecology and Evolution, Institute of Biological Sciences, Federal University of Minas Gerais, Belo Horizonte, Brazil; ^3^ Promove College of Technology, Belo Horizonte, Brazil

**Keywords:** *Leishmania*, chemotherapy, drug resistance, systems biology, systems parasitology, molecular networks

## Abstract

Neglected Tropical Diseases include a broad range of pathogens, hosts, and vectors, which represent evolving complex systems. Leishmaniasis, caused by different *Leishmania* species and transmitted to humans by sandflies, are among such diseases. *Leishmania* and other Trypanosomatidae display some peculiar features, which make them a complex system to study. Leishmaniasis chemotherapy is limited due to high toxicity of available drugs, long-term treatment protocols, and occurrence of drug resistant parasite strains. Systems biology studies the interactions and behavior of complex biological processes and may improve knowledge of *Leishmania* drug resistance. System-level studies to understand *Leishmania* biology have been challenging mainly because of its unusual molecular features. Networks integrating the biochemical and biological pathways involved in drug resistance have been reported in literature. Antioxidant defense enzymes have been identified as potential drug targets against leishmaniasis. These and other biomarkers might be studied from the perspective of systems biology and systems parasitology opening new frontiers for drug development and treatment of leishmaniasis and other diseases. Our main goals include: 1) Summarize current advances in *Leishmania* research focused on chemotherapy and drug resistance. 2) Share our viewpoint on the application of systems biology to *Leishmania* studies. 3) Provide insights and directions for future investigation.

## Introduction

**Figure 1:**
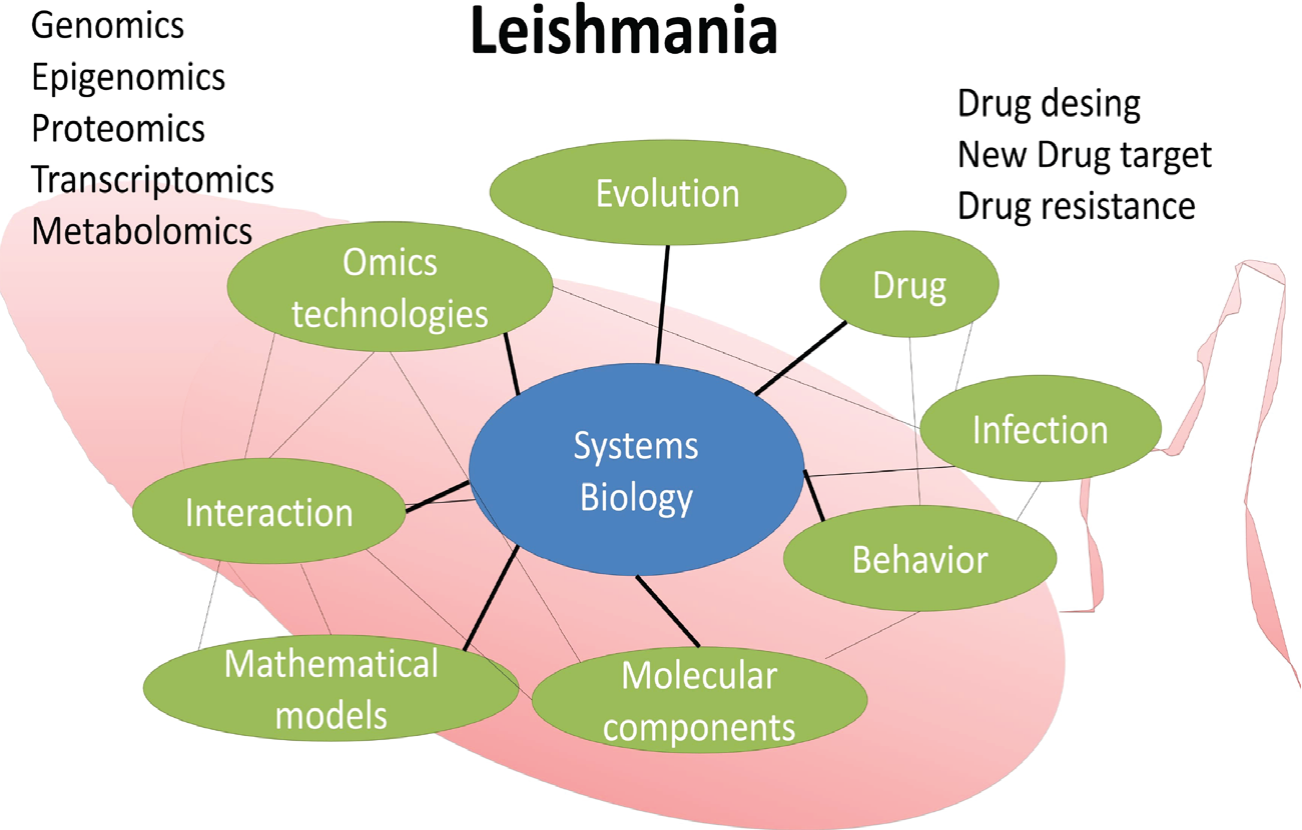
*Leishmania* systems biology. Some components of a systems biology study aiming at identifying molecular targets for drug design and development.

*Leishmania* is a complex biological system in itself. In the lack of an effective vaccine, human treatment relies on chemotherapy since the early 1920’s. Drug resistance of parasite strains adds a layer of complexity to this public health issue. Systems biology, which access interactions and behavior of complex biological processes, may improve knowledge of *Leishmania* drug resistance. **Figure 1** shows the major components of *Leishmania* systems biology discussed in the present work.

Here we present our perspective by providing a viewpoint on some specific areas of investigation as well as current advances and future directions. For this purpose, this article is organized into the following topics: *Leishmania* and leishmaniasis; Leishmaniasis treatment; Chemotherapy and antioxidant defense enzymes; Systems biology: concepts and applications; *Leishmania* systems biology; and Conclusions and future directions.

## *Leishmania* and Leishmaniasis

Leishmaniasis are among such diseases currently affecting 12 million people worldwide and presenting an incidence of 0.7-1.0 million new cases annually from nearly 100 endemic countries (WHO, 2021). Leishmaniasis are caused by over 21 different species of unicellular protozoan parasites of the genus *Leishmania* (Trypanosomatidae), which are transmitted to humans by infected female phlebotomine sandflies (Phlebotominae).

Some peculiar features are described for *Leishmania* and other Trypanosomatidae such as their kinetoplast, mitochondrial DNA editing (Simpson and Shaw, 1989; Ibrahim et al., 2008), glycosomes (Michels et al., 2006), polycistronic transcription (Martínez-Calvillo et al., 2003), trans-splicing (Boothroyd and Cross, 1982; Liang et al., 2003), GPI-anchored proteins (Mensa-Wilmot et al., 1999), and absence of promoter-mediated regulation of nuclear genes (Stefano et al., 2017). *Leishmania* species present a remarkable degree of conservation in gene content and architecture (synteny) according to their evolutionary divergence (Peacock, 2007; Lynn and McMaster, 2008; Real et al., 2013).

## Leishmaniasis Treatment

There is no human vaccine available against *Leishmania* infection and control is based mainly on chemotherapy using a few drugs currently available. Leishmaniasis chemotherapy presents several issues, such as high drug toxicity, long treatment protocols, and the occurrence of drug resistant parasite strains.

It is important to highlight that drug resistance and therapeutic failure are not synonymous. Therapeutic failure encompasses factors related to the host (e.g. patient immune system and genetic factors), infectious agent (e.g., drug resistance, virulence, and pathogenic profiles of parasite species or strains), drugs (e.g. pharmacodynamics/pharmacokinetics), chemotherapeutic protocol, etc. (Ponte-Sucre et al., 2017).

Nevertheless, isolate’s drug resistance status is the first indication for therapeutic choice. *Leishmania* drug resistance threatens the prevention and treatment of infections. Literature shows that the mechanism of drug resistance in *Leishmania* involves different metabolic pathways including several molecular markers. However, little is known about the biochemical mechanisms underlying drug resistance in field isolates of this parasite. System biology approaches are very important to elucidate drug resistance mechanisms and identify new molecular markers and targets for drug development against leishmaniasis.

### Pentavalent Antimonials

Pentavalent antimonials (e.g. sodium stibogluconate and meglumine antimoniate) have been used as the first-line treatment in many countries (Croft et al., 2006). Their mode of action is still not completely understood. It has been reported that antimony inhibits macromolecule biosynthesis in amastigotes, possibly *via* the inhibition of glycolysis and fatty acid oxidation (Berman et al., 1987), changing the thiol redox potential (Wyllie et al., 2004), DNA fragmentation, and apoptosis (Sereno et al., 2001; Sudhandiran and Shaha, 2003).

Treatment failure with pentavalent antimony (Sb^V^) has been reported in Bihar (India), where more than 60% of patients with visceral leishmaniasis (VL) are unresponsive to this drug (Sundar, 2001). An epidemiological survey in this region suggested that arsenic-contaminated groundwater may also be associated with the treatment failure using Sb^V^ (Perry et al., 2015). Different antimony-resistance mechanisms have been described including decreased antimony cellular entry, decreased drug reduction/activation, increased antimony efflux, and sequestration of the metal-thiol conjugate into vesicular membranes of *Leishmania* (Croft et al., 2006).

Some of these mechanisms were described in both experimental and clinical resistance to Sb^V^. Several ATP-binding cassette (ABC) transporters have been involved in Sb^V^ resistance. PGP/MRPA (ABCC3) was the first one described to be responsible for clinical resistance to Sb^V^ in *L. donovani* (Mittal et al., 2007; Mukherjee et al., 2007). Other mechanisms involved in Sb^V^ resistance in *L. donovani* include decreased drug uptake through inactivation of the aquaglyceroporin (AQP1) transporter (Mandal et al., 2010). AQP1 mutations are associated with a high level of antimony clinical resistance in *L. donovani* (Potvin et al., 2020).

Comparative proteomic and phosphoproteomic analyses of antimony trivalente (Sb^III^)-resistant (R) susceptible (S) *L. braziliensis* lines identified several potential candidates for biochemical or signaling networks associated with the antimony resistance in this parasite (Matrangolo et al., 2013; Moreira et al., 2015). Proteomic and genomic analyses of Sb^III^-resistant *L. infantum* mutants identified MRPA as a biomarker and suggested the involvement of chromosome number variations, specific gene amplifications, and SNPs as important features of antimony resistance (Brotherton et al., 2013). The transcriptomic profile showed that many pathways upregulated in *L. infantum* antimony-resistant lines are associated with protein phosphorylation, microtubule-based movement, protein ubiquitination, stress response, regulation of membrane lipid distribution, proteins involved in RNA metabolism, and other important metabolic pathways (Andrade et al., 2020). Together, these results show that the mechanism of antimony-resistance in *Leishmania* is complex and multifactorial, identifying several candidate genes that may be further evaluated as molecular targets for chemotherapy of leishmaniasis.

Several groups have used proteomic approaches for understanding the mechanisms of clinical resistance to antimony using Sb^V^-resistant *L. donovani* isolates (Vergnes et al., 2007; Kumar et al., 2010; Biyani et al., 2011). These studies showed that the SbV-resistant *L. donovani* isolates have upregulated proteins of different metabolic pathways including glycolysis, gluconeogenesis, oxidative stress, and detoxification. Some of them include: ABC transporter, HSP-83, HSP-70, GPI protein transamidase, enolase, carboxypeptidase, among others.

Studies also demonstrated that the mechanism of antimony-resistance differs among the *Leishmania* species analyzed. A comparative proteomic analysis of Sb^III^-susceptible and resistant lines of *L. braziliensis* (LbWTS and LbSbR) and *L. infantum* (LiWTS and LiSbR) showed that 71.4% of protein spots with differential abundance identified were different between both species (Matrangolo et al., 2013). Only 28.6% of protein spots were common between them. Western blotting analysis confirmed the proteomic data results. For instance, the expression of pteridine reductase was higher in the LbSbR line compared to its susceptible counterpart LbWTS. However, the expression level of the PTR1 protein was similar between both *L. infantum* lines (Matrangolo et al., 2013). Functional analysis confirmed that pteridine reductase is associated with the antimony-resistance phenotype in *L. braziliensis*, but not in *L. infantum* (Moreira et al., 2016).

### Amphotericin, Miltefosine, and Paromomycin

Amphotericin has shown efficacy for the VL treatment (Balasegaram et al., 2012). This is a polyene antibiotic that targets ergosterol, the major parasite membrane sterol. Liposomal amphotericin B shows lower toxicity compared to amphotericin B deoxycholate; however, it has a high cost. Amphotericin-resistant *L. donovani* lines selected *in vitro* displayed changes in drug-binding affinity to the plasma membrane as a result of a modified sterol composition (Mbongo et al., 1998). Treatment failure with amphotericin B has now been reported in India, where this drug has become the first-line option in areas where refractoriness to antimony is widespread (Purkait et al., 2012).

Miltefosine (hexadecylphosphocholine) is a phosphatidylcholine analogue initially developed as an antineoplastic drug shown to be very effective for the VL treatment in India (Sundar et al., 2002). This is the first and only drug administered orally against leishmaniasis. Miltefosine interferes in cell membrane composition by inhibiting phospholipid metabolism (Rakotomanga et al., 2007). The main mechanism of experimental resistance observed is associated with a significant reduction in miltefosine internalization (reduced uptake or increased efflux). Mutations or deletions in the miltefosine translocation process in *L*. *donovani* are associated with miltefosine resistance in both *in vitro* and *in vivo* assays (Perez-Victoria et al., 2006; Seifert et al., 2007). MT and/or Ros3 have also been associated with miltefosine-resistant phenotype in clinical isolates from leishmaniasis patients (Mondelaers et al., 2016; Srivastava et al., 2017).

Paromomycin is an aminoglycoside antibiotic that changes in the parasite protein synthesis, lipid metabolism, and mitochondrial activity (Maarouf et al., 1995; Maarouf et al., 1997). Clinical trials carried on in India indicated that paromomycin was effective in the VL treatment (Sundar et al., 2007). In contrast, a lower cure rate was not found in East Africa (Hailu et al., 2010). Paromomycin-resistant parasites selected *in vitro* showed a decreased drug accumulation (Bhandari et al., 2014). Differences in paromomycin susceptibility have been observed in different *Leishmania* species and clinical isolates (Prajapati et al., 2012).

## Chemotherapy and Antioxidant Defense Enzymes

Trypanosomatidae antioxidant defense has been indicated as a potential target for chemotherapy based on their mechanism for trypanothione-dependent detoxification of peroxides, which differs from vertebrates. In this system, the thiol trypanothione maintains the reduced intracellular environment by the action of a trypanothione reductase (Turrens, 2004). Other enzymes participate in the enzymatic cascade.

Superoxide dismutase removes the excess of superoxide radicals by converting them to oxygen and hydrogen peroxide. Besides, tryparedoxin peroxidase and ascorbate peroxidase metabolize hydrogen peroxide into water molecules (Turrens, 2004). In order to investigate these enzymes in the antimony-resistance phenotype, *L. braziliensis* and *L. infantum* mutant lines overexpressing them were obtained (Andrade and Murta, 2014; Tessarollo et al., 2015; Moreira et al., 2018).

Results showed that the overexpression of iron superoxide dismutase-A (Tessarollo et al., 2015), tryparedoxin peroxidase (Andrade and Murta, 2014), or ascorbate peroxidase (Moreira et al., 2018) are involved in the Sb^III^-resistance phenotype in *L. braziliensis*. However, only iron superoxide dismutase-A plays a key function in maintaining the antimony resistance in the *L. infantum* line analyzed, while the other two enzymes are not directly associated with such phenotype. These results corroborate once again that the mechanism of antimony resistance differs among the *Leishmania* species.

Drug repositioning is an effective strategy to find new applications for existing drugs (Andrade-Neto et al., 2018; Silva et al., 2021). Thus, drugs and/or compounds that interact with different proteins involved in important metabolic pathways in *Leishmania* were searched. The ascorbate peroxidase sequence of *Leishmania* was used to seek possible drugs against this enzyme. This search returned the antibacterial agent Isoniazid, a synthetic derivative of isonicotinic acid used in tuberculosis treatment.

Results demonstrated that overexpression of ascorbate peroxidase confers resistance to Isoniazid (Moreira et al., 2018). Surprisingly, Isoniazid raised the antileishmanial effect of Sb^III^, mainly against *L. braziliensis* clones overexpressing ascorbate peroxidase. Such drug combination might be a good strategy to be considered in leishmaniasis chemotherapy.

## Systems Biology: Concepts and Applications

The origin of systems biology is still under debate among scientists, with some claiming that it was first applied by Norbert Wiener and Erwin Schrödinger or Claude Bernard around 90 and 150 years ago, respectively (cf. Saks et al., 2009). Despite different viewpoints, some agree that systems biology was first coined in the 1960s, when theoretical biologists began creating computer-run mathematical models of biological systems (Noble, 1960).

In our view, systems biology is the study of the interactions and behavior of complex biological processes based on their molecular constituents. The applied analytical approach focuses on the quantitative measurement of biological processes, mathematical modeling, and reconstruction with the aim of bringing to light the transfer of information resulting from the integration of biological data (Kirschner, 2005, Noble and Bernard, 2008, Breitling, 2010).

Systems biology is interdisciplinary and includes a wide range of data from *in vivo*, *in vitro*, *in situ*, and *in silico* studies. Ideally, *in silico* studies would be integrated and validated by other data sources especially when applicable outcomes are aimed (Butcher et al., 2004; Dunn et al., 2010; Bora and Jha, 2019).

Currently, mathematical models have been extensively used to understand biological processes in life sciences, including but not restricted to the analysis of genomics, proteomics, metabolomics, and epigenomics of a broad range of taxa (Zhao and Li, 2017; Cheng and Leung, 2018; Djordjevic et al., 2019).

Considering the complex parasite biology, the study criteria are crucial for choosing the dataset to be analyzed, taking into account the number of samples, amount of noise, experimental design, etc. Together, these criteria interfere in the network construction and downstream analyzes. The statistical network inference method, type of interaction structure (scale-free, random, and small-world), and error measurement (global and local) are also relevant.

Because of the complexity of biological systems, it is important to understand the interactions among genotype, phenotype, and environment. Systems biology addresses such aspects by applying quantitative measurement, mathematical modeling, and interdisciplinary studies including ecology and evolutionary biology (Kirschner, 2005; Medina, 2005).

By using a system biology approach, a large number of non-linear molecular interactions can be explored, such as post-transcriptional or post-translational modifications, metabolic effects, and protein recruitment dynamics in different cellular compartments. The idea is to go beyond the simplistic model of gene role determination and its phenotypic effect (Likić et al., 2010).

## *Leishmania* Systems Biology

Efforts of drug repositioning and development of new drugs require systems biology approaches to understand the genetic basis of diseases including leishmaniasis. An essential aspect of systems biology in drug discovery is the identification of potential drug targets considering the presence of multiple genes and proteins involved (Kunkel, 2006; Chavali et al., 2008; Sharma et al., 2017). In parasites, this might be understood from signaling pathways in which essential proteins participate (Sharma et al., 2017). In addition to derive novel biological hypotheses about molecular interactions involved in drug resistance, such networks may provide information to support functional prediction of genes and proteins. Currently, a huge number of protein coding genes from sequencing projects are annotated with hypothetical, predicted, or unknown functions.

The so-called omics technologies have been the driving force behind systems biology (Silva et al., 2012; Moreira et al., 2015). These technologies include genomics, transcriptomics, proteomics, and among others applied to the study of a broad range of taxa including *Leishmania* (**Figure 1**). This multidirectional and interdisciplinary integration will certainly provide experimental outcomes with impact in public health.

The divide-and-conquer approach, in which a big problem is recursively breaking down into sub-problems of the same or related type simple enough to be solved, is a robust strategy to be implemented. Among the numerous fields (sub-problems) in which systems biology would play a crucial role, we highlight the issue of drug resistance in *Leishmania* treatment (Ponte-Sucre et al., 2017).

The inference of gene regulatory networks is just a “blueprint” in the discovery of new interactions among biological entities of the drug resistance in *Leishmania* and other taxa. Here we provide an overview of some components of a systems biology study aiming at identifying molecular targets for drug design and development in *Leishmania* (**Figure 1**).

The genome-scale metabolic model of *L. donovani* supported functional annotation for hypothetical or erroneously annotated genes by comparing results with experimental data (Rezende et al., 2012; Sharma et al., 2017). In addition to annotation, authors have predicted molecular networks for *Leishmania* and other Trypanosomatidae (Rezende et al., 2012; Vasconcelos et al., 2018).

System biology studies of pathway modeling may be able to identify pathways associated with mechanisms of drug resistance in *Leishmania* (Brito et al., 2017; Ponte-Sucre et al., 2017). Results of *in vitro* approaches for the identification of genes or proteins associated with drug resistance should be integrated with *in silico* studies and used for validation of the omics strategies (Kunkel, 2006). Combined drug and vaccine therapy can successfully treat leishmaniasis patients, but there are still several side effects and a high cost involved (Ghorbani and Farhoudi, 2017).

In the case of the pentavalent antimonials, a network integrating biochemical and biological pathways is reported (Ponte-Sucre et al., 2017). For instance, the ABC transport pathway is involved in drug efflux and therefore with drug resistance (Coelho and Cotrim, 2013). Aquaglyceroporin overexpression or deletion is also associated with resistance (Marquis et al., 2005). Gamma-glutamylcysteine synthetase may protect against oxidative stress and Sb^V^ (Mukherjee et al., 2009). Reduction of Sb^V^ to Sb^III^ is involved in drug activity and internalization as well as glycolysis inhibition of fatty acid oxidation (Berman et al., 1987; Roychoudhury and Ali, 2008). Trypanothione and glutathione regulate the intracellular thiol redox balance and participate in the chemical and oxidative stress defense (Croft et al., 2006; Singh, 2006; Maltezou, 2010). Tryparedoxin peroxidase from a complex redox cascade and its overexpression is linked to resistance (Wyllie et al., 2008; Andrade and Murta, 2014). Zinc finger domains are associated with drug resistance due to the ability of Sb^III^ to compete with Zn^II^ and the modulation of the pharmacological action of antimonials (Frézard et al., 2012).

One possible approach is the integration of public available RNAseq data depicting the resistance phenomena in gene regulatory networks (Andrade et al., 2020). Such approach has demonstrated how genes interact with each other and how changes in their expression levels may result, for example, in different immunological responses promoting distinct disease outcomes including leishmaniasis (Mol et al., 2018).

The resulting association among specific transcriptional states of all genes involved in drug resistance will represent a key tool for the study and modeling of this complex biological process. Altogether, these studies have the potential to lead the identification of better drug targets and markers for pathogenesis.

## Conclusions and Future Directions

Computational modeling of the molecular components of drug resistance in *Leishmania* through the biophysicochemical monitoring of genes and proteins involved in the processes is important. Integrating metabolic and signaling pathways is crucial to reveal the correlations among molecular functions and physiological processes shedding light on a broad understanding of the drug resistance phenomena.

We believe that in a near future, neither the understanding of Trypanosomatidae biology nor their drug resistance phenomena will be conceivable without studying molecular networks. In this context, protein-protein interactions and gene regulatory networks represent a practical embodiment of systems biology.

Biomarkers involved in drug resistance might be studied into more details from the systems biology perspective. Altogether, these studies could contribute to a better understanding of parasite biology and drug resistance mechanisms. Moreover, this approach will improve the knowledge of systems parasitology and open new frontiers in the identification of new molecular targets for drug development and treatment of leishmaniasis and other diseases.

## Data Availability Statement

The original contributions presented in the study are included in the article/supplementary material. Further inquiries can be directed to the corresponding author.

## Author Contributions

LN: designed and coordinated this work. JR, SM, and LN: wrote and revised the manuscript. EH and JH: collected data, wrote, revised the manuscript, and developed the artwork (figure). All authors contributed to the article and approved the submitted version.

## Funding

This work was supported by the FIOCRUZ Innovation Promotion Program (INOVA FIOCRUZ; JR: VPPIS-001-FIO-18-12, VPPIS-001-FIO-18-8, VPPIS-005-FIO-20-2-36, and VPPCB-005-FIO-20-2-42. SMFM: VPPCB-007-FIO-18-2-94), Institut Pasteur/FIOCRUZ (SM: no grant number), Minas Gerais Research Funding Foundation (FAPEMIG; SM: CBB-PPM 00610/15), and National Council for Scientific and Technological Development (CNPq; JR: 310104/2018-1. SMFM: 304158/2019-4). Graduate student fellowships were provided by the Higher Education Improvement Coordination (CAPES; EH: 88887.502799/2020-00) and FIOCRUZ Vice-Presidency of Education, Information and Communication (FIOCRUZ/VPEIC; JH: no process number).

## Conflict of Interest

The authors declare that the research was conducted in the absence of any commercial or financial relationships that could be construed as a potential conflict of interest.
